# Factors affecting first month adherence due to antiretroviral therapy among HIV-positive adults at Felege Hiwot Teaching and Specialized Hospital, north-western Ethiopia; a prospective study

**DOI:** 10.1186/s12879-018-2977-0

**Published:** 2018-02-20

**Authors:** Awoke Seyoum Tegegne, Principal Ndlovu, Temesgen Zewotir

**Affiliations:** 1 Deparment of Statistics, Bahir Dar University, Bahir Dar, Ethiopia; 2 Department of Statistics, Unisa, Pretoria, South Africa; 3 School of Mathematics, Statistics and Computer Science, University of Kwazulu Natal, Durban, South Africa

**Keywords:** Adherence, HAART, Logistic regression, Longevity, Prospective study, Treatment failure

## Abstract

**Background:**

Non-adherence to Highly Active Antiretroviral Therapy (HAART) is one of the factors for treatment failure in human immunodeficiency virus (HIV) infected patients in developing countries. The main objective of this study was to identify factors for treatment failure among adult HIV patients based on the assessment of first month adherence in the study area.

**Methods:**

The study was conducted using secondary data from antiretroviral unit at Felege Hiwot Teaching and Specialized Hospital. A prospective study was undertaken on 792 randomly selected adult HIV positive patients who have started HAART. The variable of interest, adherence to HAART was categorized as non-adherence if a patient had taken less than 95% of the prescribed medication and this was measured using pill counts. Descriptive statistics, Chi-square tests of association, independent samples t-test and binary logistic regression were used for data analysis.

**Results:**

In first month therapy, 68.2% of the patients belong to adherence group to HAART. As age increases, a patient without cell phone was less likely to be adherent to HAART as compared to patients with cell phone (AOR = 0.661, 95% CI: (0.243, 0.964)). Compared to urban patients, rural patients were less likely to adhere to HAART (AOR = 0.995, 95% CI: (0.403, 0.999)). A patient who did not disclose his/her disease to families or communities had less probability to be adherent to HAART (AOR = 0.325, 95% CI: (0.01, 0.64)). Similarly, a patient who did not get social support (AOR = 0.42, 95% CI: (0,021, 0.473)) had less probability of adherence to HAART. The main reasons for patients to be non-adherent were forgetfulness, side effects, feeling sick and running out of medication.

**Conclusion:**

This study indentified certain groups of patients who are at higher risk and who need counseling. Such groups should be targeted and tailored for improvement of adherence to HAART among HIV positive adults. The health care providers should advise the community to provide social support to HIV positive patients whenever their disease is disclosed. On the other hand, patients should disclose their disease to community to get integrated supports. HIV infected patients who are directed to start HAART should adhere the prescribed medication. For the adherence to be effective, patients who have cell phone should use them as reminder to take pills on time.

## Background

The greatest health-related problem in the world, especially in Sub Saharan countries, is HIV/AIDS. At the end of 2009, an estimated number of 33.3 million people were living with HIV. Among these, 2 million were newly infected and 1.8 million died due to HIV/AIDS [1]. Out of the total HIV infected patients, Sub-Saharan countries account for 22.5 million people (68%) [1]. An estimated number of 753,100 people were living with HIV/AIDS in Ethiopia with national prevalence rate of 1.5% in 2011 and had been predicted to decrease to 1.1% in 2015 [2]. Although the national HIV prevalence rate was 1.5%, Amhara region, one of the eleven regions in the country, had a prevalence rate of 1.6%, which is a serious case as compared to the national one [3]. Hence, the region needs special attention to decrease the prevalence of HIV/AIDS and to reduce HIV related problems like non-adherence to HAART. HAART has dramatically reduced morbidity and mortality that could result from HIV/AIDS [4]. Next to CD4 cell count, adherence to HAART is the second strongest predictor of progression rate of HIV/AIDS and death [5].

Non-adherence to medication is a wide spread among seriously infected patients in clinical trials and practice [4]. Starting HAART for lately diagnosed HIV-infected individuals had increased adherence challenges for both the patients and for the HAART program to be effective [6]. Studies reported conflicting evidences about the association of adherence to HAART and its predictors. Some researchers found that socio-demographic variables have statistically significant effect over adherence to HAART, while the others found that there is no association among them [7–9]. Recent studies on the impact of adherence to HAART treatment outcomes in Sub-Saharan countries indicate that there is an improved immunologic response and clinical outcomes among HIV patients with optimal adherence [10]. Even after a month of therapy, a patient with optimal adhrence indicated that there are significant treatment responses like CD4 cell count change, viral suppersion and weight gain [10]. Viral load suppression is one of the most reliable indicators of adherence performance [11]. The efficacy of HAART depends on strict adherence to the regimen, but many factors have been identified for non-adherence [12]. Non-adherence is one of the most contributing factors in treatment failure and is potentially amenable to intervention [12]. Optimal adherence to HAART has a positive association with an improved long-term treatment outcomes [10]. Adherence to HAART is crucial to ensure viral suppression and minimize the risk of disease progression and drug resistance. However, measuring adherence accurately is not a simple task. That is why a number of conflicting reports are available from different studies conducted on adherence to HAART [13]. Because of methodological difficulties, it is not surprising that a large number of factors have been reported as predictors of adherence to HAART. Some optimistic literatures [14, 15] have over-emphasized adherent patients based on selective publications [16]. Such literatures might be biased towards highly motivated patients with early access to limited therapy [17]. On the other hand, the health professionals at the initial time of HAART can not accurately predict individuals at risk of optimal adherence to HAART. This is because, there is no prior information about each patient related to optimal adherence to HAART or there is no adherence practice before the first month [18]. Therefore, developing tools that assist clinicians in determining factors related to non-adherence to HAART at the initial period is important to intervene into HAART program at early stage. For treatment progress and viral replication, the HIV patients should adhere at least 95% of all the prescribed medication [10]. Adherence to HAART can be influenced by a number of factors like social situation, clinical condition, the prescribed regimen, and patient-care provider relationship [11]. Optimal adherence to HAART is also affected by the individual factors such as drug use, alcohol use and age of patients. Medication regimen includes dosing complexity, number of pills and food requirement and the system of the care [6]. It is critical that each patient should understand about the information on predictors of HIV disease associated with optimal adherence to HAART [11]. One of the problems in assessing the behavioral trends and predictors about the prevalence of HIV is lack of real information [19]. Similarly, there is limited information available from the study area about initial predictors of adherence to HAART. Available research in Ethiopia makes our understanding about factors associated with initial adherence to HAART limited; and related literatures in the study area are remarkably scarce. Few investigators, who did research about adherence to HAART, have discrepancies on their findings. Understanding of predictors of adherence to HAART in developing countries is a forefront issue in the study area where little is known in the scaling up of the progress of HAART implementation. Addressing issues related to adherence to HAART may provide valuable information about which patients are at risk for non-adherence and how adherence might be improved. Therefore, the purpose of this study was to assess whether or not global experience works at Felege Hiwot Teaching and Specialized Hospital, north-west Ethiopia; that is, to identify factors affecting adherence on the onset of HAART in the study area. The result of first month adherence study helps improve first month optimal adherence to HAART and reduce the number of patients that could be referred to more advanced and expensive second-line regimens. The other importance of this study is in developing tools that assist clinicians in long term follow-ups of patients and further communicating and counseling strategies in relation to HAART treatment.

## Methods

### Sampling procedures

Before selecting HIV/AIDS infected patients to be included in the sample, the list of HIV/AIDS patients, who have been taking HAART, was collected from ART section of Felege Hiwot Teaching and Specialized Hospital. A sampling frame was prepared in collaboration with a health care service providers and the ART section in the hospital. Stratified random sampling technique was applied in sample unit selection procedure considering their residence areas as strata.

### Study site, population and design

A prospective study was conducted in antiretroviral therapy unit at Felege Hiwot Teaching and Specialized Hospital in the initial month of HAART. A total of 6036 PLWHIV were receiving health care service. Among the total patients, 2356 (39%) were on HAART. The study used secondary data obtained from charts of randomly selected 792 adult HIV/AIDS patients at Felege-Hiwot Teaching and Specialized Hospital.

### Variables included in the model

The dependent variable was adherence to HAART measured using pill counts and self-reported data. The independent variables included under current investigation were socio-economic characteristics (level of education, age, disclosure level of the disease and social support, gender, marital status, memory aids/ownership of cell phone and level of household income) and clinical characteristics (weight, baseline CD4 cell count, WHO stages).

### Measurement

To measure adherence to HAART; there were two approaches: pills count approach and patients’ self reported data recorded by the health staff. The first approach helped compute adherence rate and the second one helped in identifying associated factors with HAART adherence. At initial time of HAART, patients took pills from the hospital, calculated for one week (7 days) and they were oriented to visit the hospital every week for one month to refill bottles. Patients were also oriented to take pills after meal. Dose adherence/Pill counts were also conducted by health staff. A health staff categorized a patient as adherent if a patient took at least 95% of the prescribed pills; otherwise, he/she is non-adherent. To assess whether patients took pills on time as directed by care provider, the study considered self-reported time adherence; and, in this regard, a person is adherent if he/she claims to follow time scheduling instruction. To assess the importance of diet on adherence to HAART, self-reported food adherence was also included in the investigation. This self-reported food adherence helped check whether patients follow dietary instruction by care providers. The self-reported adherences (food and time) were collected using a pretested structured questionnaire which consists of socio-demographic characteristics, psychological attributes and health care systems such as dosing schedules, frequency, side effects (depression/headache) and dietary demands.

Patients were oriented to return bottle of pills including unused/remaining pills (if any) to the hospital at the end of each week. The medical staff counted unused pills and computed adherence to HAART using pill counts and applied the formula, $\frac{total\ pills\ used}{total\ pills\ prescribed\ for\  one\  week}\ast 100\%$ and categorize patients as adherent or non-adherent. Self-reported time and food adherences were recorded for each patient in their individual documents/charts. Patients recalled back 7-days adherence performance regarded to time and food. The interview was done every week to investigate the variation in degrees of association between factors with adherence at these periods.

### Analysis

Data collected at Felege Hiwot Teaching and Specialized Hospital were cleaned, coded, entered and analyzed. The reliability of self-reported data was assessed using Crombanch’s alpha. A cut-off value of 0.71 was used to indicate acceptable internal consistency [20]. Bivariate logistic regression model was conducted to assess predictors of adherence to HAART. In all assessments, explanatory variables associated with adherence to HAART in bivariate case with *p*-values ≤0.25 were included in multi-variable analysis. The model was evaluated with forward and backward elimination process considering p-value of 0.2 and 0.1 respectively [20]. The statistical methods such as descriptive statistics, Pearson’s Chi-square test, t-test and logistic regression models were used to analyze the data using SPSS version 23 with the level of significance α = 0.05. The deviance and Pearson’s Chi-square were used to compare alternative models during model selections. The selected model was the one with the smallest deviance. Change in the deviance was used to measure the extent to which the fit of the model improved when additional variables were included.

## Results

Among baseline characteristics, patients who disclosed the disease to family members reported that they received social support from families, friends, clubs and neighborhoods in each visiting time. Applying HIV/AIDS stigma scale, that has been conducted by health staff (Crombanch’s alpha = 0.2) at each reporting time showed that, on average among patients who disclosed their disease, 543 (94.4%) of the study subjects got social-support and the rest were stigmatized. An average number of 52 (6.6%) patients reported that they were active substance users. Depression inventory was conducted using Beck’s depression inventory scale with a cut-off point ten at each of the assessment periods. With this regard, on average 320 (40.4%) of the study subjects were depressed. Side effects like skin rash, gastritis and headache were experienced on an average of 250 (31.6%) patients. The rate of self-reported adherence was mainly based on whether participants had followed time and meal instructions to take pills. From the self-reported data, it was found that 450 (56.8%) of the study subjects reported that they followed instruction related to food ordered by health professionals and 389 (49.1%) of the study units made practical for time adherence restrictions oriented by the health care service providers. The reasons for non- adherence on the prescribed medication were summarized from self-reported and recorded data; and on average, it was found that 52.5% of the patients forgot to take pills, 12.5% felt sick, 8.5% felt drowsy and 6.7% ran out of medication. Generally, the baseline characteristics of participants included in the analysis are presented in Table 1. The median age of patients was 36 years (IQR: 28–48). Of the participants, 50.6% were females, 40.1% were living in rural areas, and 55.2% were living without partners. Among the patients, 27.4% did not disclose the disease to family members living together and only 68.18% of the patients were adherent to HAART in the first month treatment. About 50% of the patients had attended their secondary education and 50% of them had cell phone. The average baseline CD4 cell count for all patients was 134 cells/mm3 (IQR: 113–180) and the average CD4 cell count change for the first month was 15.9 cells/mm3 (IQR: 9–26).

**Table 1:** Baseline socio-demographic and clinical characteristics of the HAART patients (*n* = 792)

Variables	Characteristics	Median (IQR)	*n* (%)
Weight	Base line weight in K.G.	62 (58–70)	
Base line CD4	Baseline CD cell count	134 (113–180)	
Age	Age in years	36 (28–48)	
1st month CD4 count change	First month CD4 cell count change	15.9 (12–26)	
Sex	Male		391(49.4%)
	Female		401(50.6%)
Level of education.	No educ.		160 (20.2%)
	Primary		205 (25.9%)
	Secondary		273(34.5%)
	Tertiary		154 (19.4%)
Residence area	Urban		468 (59.1%)
	Rural		324 (40.1%)
Marital status	Living with partner		355 (44.8%)
	Living without Partner		437(55.2%)
Level of income	Low income (< 500 ETB per month)		355 (44.8%)
	Middle income (5001–999 ETB per month)		346 (43.7%)
	High income (≥1000ETB per month)		91(11.5%)
WHO stages	Stage I		101(12.8%)
	Stage II		258 (32.6%)
	Stage III		199 (25.1%)
	Stage IV		234 (29.5%)
Disclosure level of disease to families	Yes		575 (72.6%)
	No		217 (27.4%)
Owner of cell phone/memory aids	Yes		400 (50.5%)
	No		392 (49.5%)
First month dose adherence	Yes		392 (49.5%)
	No		400 (50.5%)
First month food adherent	Yes		385 (48.6%)
	No		407 (51.4%)
First month time adherent	Yes		350 (44.2%)
	No		442 (55.8%)
First month adherence to HAART	Yes		540 (68.18%)
	No		252 (31.82%)
Social support	Yes		500 (63%)
	No		292 (37%)

All included and excluded sample units in this cohort had almost the same baseline characteristics (age, base line CD4 cell count, weight, gender). Applying independent samples t-test for quantitative variables and proportion for categorical variables in SPSS, we had weight (means, included in the study = 57.5 kg and those excluded in the study = 59 kg, *p*-value = 0.73), and gender (male included in the study = 54.5% and excluded from the study = 53%, p-value = 0.78). The mean ages of patients in non-adherent and adherent groups were 48.1and 26.3 years, respectively. This indicated that more youngsters were optimally adhered to HAART as compared to elders. Similarly, the average changes in CD4 cell counts in first month of the treatment were 8.7 and 19.6 cells per mm3 for non-adherent and adherent patients respectively. Adherence rates for baseline characteristics of patients presented at Table 2, shows that all categories of predictors except residence area (*p*-value = 0.079) had very strong relationship with the response variable, optimal adherence to HAART. The Chi-square tests of association and their respective *p*-values are indicated in Table 2.

**Table 2:** Adherent rates by baseline characteristics of study participants

Category of predictors		Patients’ adherence rate		Total	*P*-value)
		Non-adherent *n* (%)	Adherent *n* (%)		
Residence area	Rural	300 (28.4)	114 (71.6)	324	0.079
	Urban	228 (31.4)	150 (68.6)	468	
Educational background	No education	208 (80)	52 (20)	260	0.000
	Primary	190 (69.1)	85 (30.1)	275	
	Secondary	35 (34)	68(66)	103	
	Tertiary	10 (6.5)	144 (93.5)	154	
Marital status	Living with Partner	51 (14.4)	304 (85.6)	355	0.000
	Living without partner	188 (43)	249 (57)	437	
Gender	Female	97 (24.8)	294 (75.2)	391	0.018
	Male	142 (35.4)	259 (64.6)	401	
Household income	Low income	165 (46.5)	190 (53.5)	355	< 0.001
	Middle income	116 (35.5)	230 (64.5)	346	
	High income		91 (100)	91	
Owner of cell phone	With cell phone	51 (9.4)	490 (91.6)	541	< 0.001
	Without Cell phone	60 (23.9)	191(76.1)	251	
Level of Disclosure	Disclosed the disease	18 (4.8)	357 (95.2)	375	
	Not disclosed the disease	339 (81.3)	78 (18.7)	417	< 0.001
WHO stages	Stage I	4 (4)	97 (96)	101	0.001
	Stage II	96 (37.2)	162 (62.8)	258	
	Stage III	124 (62.3)	75 (37.7)	199	
	Stage IV	163 (69.7)	71 (30.3)	234	
	Yes	120 (24)	380 (76)	500	< 0.001
Patients who got social support	No	192 (65.8)	100 (34.2)	292	

Table 2 indicates that, among patients who got social support, the majority (76%) were adherent. On the other hand, among patients who did not disclose the disease to communities and families, the majority of them (81.3%) were non-adherent to HAART. Moreover, 91% of the patients with ownership of cell phone belonged to adherent group with *p*-value < 0.001. More educated patients were categorized as adherent as compared to non-educated patients. Hence, among tertiary level educated patients, 93.5% were adherent, while 90% of non-educated patients were non-adherent with *p*-value < 0.001.

### Model selection

From the different alternatives for model selection, a model with all main and interaction terms of smallest deviance was selected. Goodness of fit of the selected model had been assessed applying Hosmer-Lemeshow statistics (*p* = 0.621) which indicated that the model was satisfactory. Influential observations were also tested with Cook’s distance statistic and the result showed that there were no influential observations. The link function and its square were also checked. The link function was appropriate and its linear predictor was significant (*p*-value = 0.002); however, its square was insignificant (*p* = 0.085). The adjusted odds ratios (AOR) and the corresponding 95% confidence interval are given in Table 3.

**Table 3:** Parameter estimation for multi variable logistic regression model on optimal adherence

Parameter	B	Standard error	AOR	95% CI for Exp(B)		*P*-value
				Lower	Upper	
(Intercept)	0.971	4.2297	2.641	1.002	3.043	0.009
Age	−0.046	0.0705	0.063	0.032	0.196	0.013*
Weight	− 0.095	0.0425	0.9101	0.837	1.098	0.055
Baseline CD4 cell count	−0.005	0.0078	0.995	0.403	0.999	0.010*
Residence (reference = urban)						
Rural	−0.186	0.3285	0.83	0.533	0.993	0.001*
Education (reference = Tertiary)						
No-education	−2.25	0.105	0.105	0.000	4.643	0.464
Primary education	−2.68	0.168	0.069	0.000	7.976	0.345
Secondary education	−1.41	0.244	0.2441	0.001	9.282	0.639
Marital status (reference = living without partner)						
Living with partner	0.782	0.4763	2.187	1.860	5.562	0.010*
Gender(reference = male)						
Female	0.011	2.0552	1.011	1.009	1.365	0.023*
Household income (reference = high income)						
Low income	−0.996	4.2249	0.37	0.001	0.64	0.024*
Middle income	−0.919	4.2249	0.399	0.063	1.871	0.994
Ownership of cell phone (reference = yes)						
No	−0.328	2.377	0.72	0.32	0.94	0.009*
Level of disclosed disease (reference = yes)						
No	−1.124	1.2463	.325	0.01	0.64	0.008*
WHO stages (ref = WHO stage 4)						
WHO stage 1	0.210	2.3145	1.233	0.009	4.054	0.933
WHO stgage 2	−0.827	1.6532	.437	0.137	1.392	0.162
WHO stage 3	−0.363	1.2351	.696	0.321	1.510	0.359
Social support (reference = yes)						
No	−0.874	1.4321	0.4173	0.021	0.743	< 0.001*
Food adherence (reference = yes)						
No	−0.921	1.236	0.398	0.086	0.563	< 0.001*
Age^a^ patients with owner of cell phone (reference = yes)						
Age^a^ no	0.013	1.3210	1.013	1.002	1.0664	0.009*
Age^a^gender(reference = male)						
Age^a^ female	−0.104	2.1024	0.90123	0.7345	0.9998	0.005*
Baseline CD4 cell count^a^ gender (reference = male)						
Female^a^ baseline CD4	−0.108	1.1200	0.89763	0.5703	0.9456	0.042*
Owner of cell phone^a^ initial CD4 cell count (reference = yes)						
Baseline CD4 cell^a^ no	0.128	0.8801	1.1427	1.0027	1.2832	0.008*

^a^Indicates significant at 95% CI

As shown in Table 3, age, baseline CD4 cell count, weight, marital status, sex, owner of cell phone and patients who did not disclose the disease to family members living together, patients with strict time and food adherence and social support had significant association with first month optimal adherence to HAART. As the age of the study unit increased, Patients were less likely to be optimal adherent to HAART. Patients with high CD4 cell count at the onset of HAART were less likely to be adherent to HAART. Similarly, patients with cell phone, female patients, patients who disclosed the disease to families and relatives and those patients who got social supports from others were more likely to be adherent to the prescribed medication directed by care providers (Refer to Table 3). As indicated in Table 3, four of the interaction effects were significantly associated with the variation of optimal adherence to HAART. The following were significant interaction effects summarized in Table 3.

- Interaction effects between age of patients and ownership of cell phone: As the age of study participants increased, the decreasing rate of optimal HAART adherence for patients with cell phone was 34% less likely as compared to patients without cell phone/memory aids (AOR = 0.661, 95% CI: (0.243,0.964); *p*-value = 0.0009)). Hence, as age of a patient increased, optimal adherence decreased but with different rate of decrease for the two groups (with and without cell phone).

**Fig. 1:**
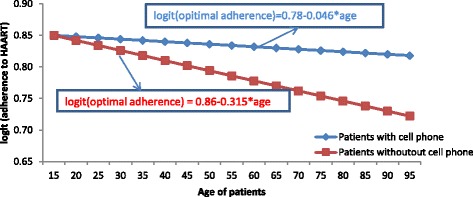
The plot of interactions effect between age and ownership of cell phone

Figure 1 indicates that as age of a patient increased, the gap between the logit of optimal adherence to HAART for patients with and without cell phone/memory aid also increased. Hence, those patients who used cell phone as reminder had better adherence level as compared to those patients who did not use cell phone as reminder.

**Fig. 2:**
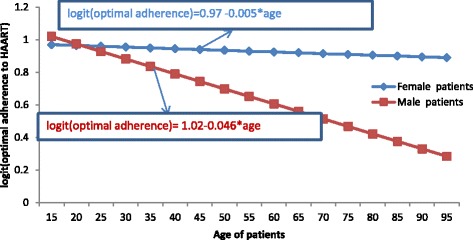
The plot of interactions effect between age and gender

b) Interaction effects between age and gender of patients: Table 3 shows that, as patients’ age increased, optimal adherence to HAART decreased but with different rates for female and male patients. Hence, as age increased by one unit, the decreasing rate of the logit of optimal adherence for females was 10% less likely than the logit of optimal adherence to HAART for male patients (AOR = 0.90, 95% C.I (0.7343, 0.9998); *p*-value = 0.005))(Refer to Fig. 2).

Figure 2 indicates that as age increased, males and females had a larger gap for the logit of adherence to HAART. Hence, as age of patients increased, all males and females had decreased their adherence to HAART. However, females had less likely to decrease adherence level as compared to males.

c) Interaction effects between baseline CD4 cell count and gender: Table 3 indicates that, optimal adherence to HAART was less likely for patients with high baseline CD4 cell count but whose laboratory results evidenced to start optimal adherence to HAART. Hence, as baseline CD4 cell count increased by one unit, the decreasing rate of the logit of adherence to HAART for females was 11% less likely than the logit of adherence to HAART for males (AOR = 0.89, 95% CI: (0.5703, 0.9456); *p*-value = 0.0042)).

**Fig. 3:**
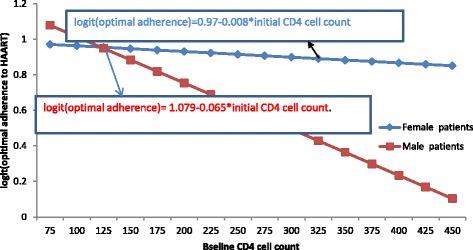
The plot of interaction effect of baseline CD4 cell count and gender

Figure 3 shows that, as baseline CD4 cell count increased, the logit of adherence for both sexes had decreased, but females had less decreasing rate for the logit of adherence to HAART as compared to males.

d) Interaction effects between owner of cell phone and baseline CD4 cell count**:** Table 3 indicates that baseline CD4 cell count had a negative significant effect on the logit of adherence to HAART. As baseline CD4 cell counts increased by one unit, the decreasing rate of the logit of optimal adherence to HAART for patients without cell phone was 14% more likely than those patients with cell phone (AOR =1.1427,95% CI: (1.0027, 1.2832); *p*-value = 0.008)).

**Fig. 4:**
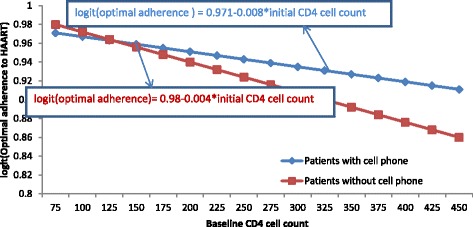
The plot of interaction effects between owner of cell phone and baseline CD4 cell count

As it is indicated in Fig. 4, as baseline CD4 cell count increased, the logit of adherence for all patients decreased. But the decreasing rate for patients with cell phone was less likely as compared to patients without cell phone.

## Discussions

The results from this one month of prospective study, the main effects like age, weight, baseline CD4 cell count, residence area, marital status, level of disclosure of the disease to families and communities, ownership of cell phone, social support and adherence to time and food had significant effect on optimal adherence to HAART. A patient enrolled with high baseline CD4 cell count had low optimal adherence to HAART as compared to those patients who had low baseline CD4 cell count. Hence, patients with high baseline CD4 cell count but who had been directed to start adherence to HAART, missed lots of follow-ups from HAAR. The reasons for greater number of missing the prescribed medications might be that healthier individuals might lack the experience of taking pills frequently. Since such patients also feel comfortable; so, they might lack interest to be strict adherent to HAART on time. This practice for HIV patients seems to have negative effect on optimal adherence [21, 22]. Patients who lack experience of severe opportunistic infections might lead to forgetfulness for taking pills on time [23]. Therefore, to reduce such forgetfulness, an intervention can be held in using memory aids such as cell phone as reminder [24]. Patients with cell phone had higher adherence performance as compared to those without cell phone. This indicates that cell phone may be used as memory aid to remind patients to take pills on time [24]. Our results seem to indicate that female patients have better adherence performance compared to males. The reason for this might be females’ experiences in taking pills for family planning/for birth control. Most of the time, females are available around their home and they can adhere strictly their prescribed medication directed by the care providers. The correlation among male patients who run out without pills, contributes for them to be non-adherent.

The results of first month study indicate that patients who were adherent to HAART got high CD4 cell count change (increase of CD4 cell count) as compared to non-adherent patients. This indicates that the correlation between good adherence and greater increase of CD4 cell count confirms the importance of interventions to improve compliance to the therapy.

Urban patients were more likely to be adherent to the prescribed medication as compared to rural patients. This might be the reason that patients at urban and catchment areas of it had better understanding on the use of adherence to HAART for longevity of their life as compared to those patients living in rural areas. The challenges in the rural community, particularly for HIV patients are lack of balanced diet, long distance from health institutions to conduct diagnosis timely at initial time, and give less attention for their health [25]. Patients who were strict on food adherence were more likely to be optimal adherent to HAART as compared to non-adherent to food. Patients who had lack of food and having hunger but initiated HAART might be considered as one of the reasons for low adherence to HAART and its response, CD4 cell count change [26]. Patients living with their partners had better HAART adherence as compared to patients living without partners. The reason for this might be that partners encourage patients to take pills on time and may support each other [27].

Similarly, patients who disclosed the disease to families living together had better HAART adherence. If families are aware of the disease, they can remind a patient to take pills on time; they may provide balanced diet so that the patients can have more resistance and develop more interest to be adherent to HAART. This study also indicates that the positive correlation between adherent patients for HAART, food, time and good progress of CD4 cell count confirms for longevity of life of patients in the HAART program and for the program to be effective. This result is supported by previous study [27].

## Conclusion

This study tried to identify certain groups that need special intervention at the initial time of HAART. Patients who started with high baseline CD4 cell count had less likely to be optimal adherent to HAART. Due attention should be paid to create awareness for non-adherent patients to be adherent to HAART which is an essential for the treatment to be effective. Rural patients, who did not take early diagnosis for HIV/AIDS, need intervention for awareness creation to come on time for such diagnosis and for the treatment to be effective. Those patients who did not adhere to time and food /meal instructions should be counseled to use memory aids like cell phone. Hence, no single intervention strategy can improve the adherence to HAART for all patients. Previous literature also supports this idea, and the assessment of key issues related to adherence to HAART in all directions can improve the longevity of patients [23].

This study is not without limitations. One limitation was that the significant interactions effects existed in the study were observed during data analysis and were not considered in data collection. Therefore, how and why these interactions affect optimal adherence, cannot be answered in this study. The issue that needs attention is the need for long-term study concerning whether or not covariates which affected adherence to HAART in one month of therapy also have effects through time. The data had been taken in one treatment site. Considering two or more sites in the investigation may have results different from these or may have additional information for these results. In addition, a further study on adherence rate and its determinants with multiple adherence measurements is recommended. This needs further investigation and can be considered as potential research gaps for future investigators.

The strength of this study lays in the fact that, it suggests interaction effects between certain characteristics of patients which did not exist in previous literatures and advantageous for future researchers to work in the area. Besides, the research tried to identify certain groups that require special attention and this helps intervene in the HAART activities to be effective which elongates the life of patients in HAART program.

## Abbreviations

AIDS: Acquired Immune deficiency Syndrome
AOR: Adjusted Odds Ratio
CD4: Classification Determinant Four
HAART: Highly Active Ant-Retroviral Therapy
HIV: Human Immune Deficiency Virus
PLWHA: Patients Living with HIV/AIDS
SPSS: Statistical Package for Social Science
WHO: World Health Organization
